# Flood-associated disease outbreaks and transmission in Southeast Asia

**DOI:** 10.3389/fmicb.2025.1694246

**Published:** 2025-10-22

**Authors:** Boonfei Tan, Patrick De Vera, Josephine Abrazaldo, Charmaine Ng

**Affiliations:** ^1^Manila Central University, Caloocan, Philippines; ^2^College of Medical Technology, Manila Central University, Caloocan, Philippines

**Keywords:** waterborne disease, floods, wastewater surveillance, ASEAN, disaster microbiology

## Abstract

Southeast Asia (SEA) is among the world’s most flood-prone regions, where climate change is intensifying rainfall and extreme weather events. Floods disrupt communities and pose risks of infectious disease by bridging human, animal, and environmental reservoirs of pathogens. These events add strain to countries with vulnerable healthcare systems and critical infrastructure. Regional platforms such as the ASEAN Coordinating Centre for Humanitarian Assistance (AHA) and the ASEAN Biodiaspora Virtual Centre provide valuable weekly updates on emerging infectious diseases that could support disaster preparedness and response by incorporating supporting epidemiological and environmental data on waterborne outbreaks. Evidence synthesized in this review shows how floods reshape pathogen persistence, transmission pathways, host–environment interactions, and antimicrobial resistance (AMR), within the SEA context. By complementing existing regional monitoring endeavors, a One Health perspective emerges as a useful lens to capture the interconnected nature of risks across human, animal, and environmental domains. Advances in wastewater and environment-based surveillance, coupled with multi-omics approaches and machine learning, create new opportunities to detect diverse pathogens, integrate complex datasets, and forecast risks with more precision. This review addresses the importance of considering pathogen transmission before, during and after flood events, framing infectious disease risks within broader ecological and socio-economic contexts. By adopting this holistic perspective within the one-health paradigm, SEA countries could strengthen preparedness and resilience strategies before disasters occur.

## 1 Introduction

Human-induced climate change has resulted in rising sea levels and an increase in severe weather events such as heatwaves, droughts, wildfires, and unpredictable fluctuations in heavy precipitation that lead to flooding (Stott et al., 2016; Zhai et al., n.d.; Mora et al., 2022). Floods have a major impact across regions globally. About 1.8 billion people (23% of the global population) are exposed to 1-in-100-year floods, with the greatest risks concentrated in low- and middle-income countries (LMIC), especially South and East Asia (Rentschler et al., 2022). Increased rainfall intensity, a consequence of climate change, puts many regions across Southeast Asia (SEA) at risk of major floods on an annual basis, particularly during the monsoon season (Chen et al., 2025; Loo et al., 2015). Of all disaster categories recorded by the ASEAN Coordinating Centre for Humanitarian Assistance on disaster management (ASEAN Coordinating Centre for Humanitarian Assistance on disaster management (AHA Centre), 2025), an ASEAN (2025) inter-governmental body responsible for disaster risk management in the region, floods are the most frequent disaster type across ASEAN Member States (AMS), accounting for 63% of all reported disaster events in this region. Floods directly threaten the livelihood of 23% of the total ASEAN population (approximately 146 million people) (AHA Centre, 2024), and cause major economic loss; with Indonesia, the Philippines, Thailand, Vietnam, Malaysia, and Myanmar, each incurring between US$3 and 30 billion annual average loss per year (ASEAN Secretariat, 2025).

In the aftermath of disasters, displacement, disruption of medical services, and damage to health and civil infrastructure are the most visible effects (Wisner and Adams, 2002). Less apparent, however, are ecosystem-level changes that may heighten health risks. From an environmental perspective, climate change and extreme weather events are increasingly recognized as drivers of contaminant mobilization, fate, and transport. Both chemical and microbial hazards can persist and move through complex pathways, creating cross-sectoral risks for ecosystems and public health (Mora et al., 2022; Bolan et al., 2024; Crawford et al., 2022; Laino and Iglesias, 2025; Noyes et al., 2025; Uwishema et al., 2023; Zitoun et al., 2024). These dynamics are particularly critical in SEA, where recurrent floods and heavy rainfall across AMS repeatedly expose populations to unsafe water. In this setting, floodwaters act as vehicles for pathogens already present at low levels in the environment, while socio-economic vulnerabilities (Rentschler et al., 2022) amplify transmission risks (Acosta-España et al., 2024). Against this backdrop, outbreaks of waterborne diseases have historically manifested after flood events, exemplified by the most recent cholera outbreak in Myanmar in 2024 (World Health Organization, 2025a). As of August 2025, several major cities in SEA were struck by massive floods, with numerous official (ADInet) and unofficial news outlets circulating reports of potential risks of outbreaks (ASEAN Coordinating Centre for Humanitarian Assistance on disaster management (AHA Centre), 2025).

Systematic reporting of flood-associated disease outbreaks across AMS remains limited. Where available, historical literature frequently focuses on leptospirosis (Sawangpol et al., 2025), diarrhea disease (Asadgol et al., 2020; De Guzman et al., 2015; Glass et al., 1984; Lopez et al., 2015; Simanjuntak et al., 2001), typhoid fever (Nga et al., 2018; Roberts et al., 2020; Alba et al., 2016; Muhammad et al., 2020; Pham Thanh et al., 2016), malaria (Memon et al., 2014; Bharati and Ganguly, 2013) and dengue (Langkulsen et al., 2020; Wibawa et al., 2024) as the most common flood associated outbreaks in this region. This gap between well-documented flood events and the sparse reporting of associated outbreaks underscores the challenge of aligning disaster monitoring with public health surveillance. Currently, the ASEAN Weekly Disaster Update (ASEAN Coordinating Centre for Humanitarian Assistance on disaster management (AHA Centre), 2025) systematically documents major flooding events to support transnational coordination; however, flood-related outbreaks are rarely captured and typically surface only through news reports or official statements. Wastewater and environment surveillance (WES), as recommended by WHO (World Health Organization, 2024) can plug this gap to better understand disease transmission and circulation patterns within populations. Linking such approaches with the ASEAN BioDiaspora Virtual Center Dashboard (ASEAN BioDiaspora Virtual Center (ABVC), 2024), which already supports surveillance of epidemic-prone diseases such as leptospirosis, influenza, COVID-19, and Mpox, could provide a stronger disaster-health surveillance system.

ASEAN’s recent Leaders’ Declaration on the One Health Initiative and Health Cluster 2’s ‘all-hazards’ program (ASEAN, 2025) recognizes that emergencies such as floods, propagate simultaneously through people, animals, and water systems; consequently, preparedness and response cannot be siloed. AHA Centre disaster intelligence and ABVC analytics further underscore the need for interoperable indicators across health, agriculture/livestock, and water–environment authorities as floods and their disease consequences traverse provinces and borders. This position is congruent with the WHO/FAO/WOAH/UNEP Quadripartite One Health Joint Plan of Action, which prioritizes integrated surveillance and WASH/food-safety protections precisely because flooding elevates risks of water- and vector-borne disease and mobilizes environmental AMR. Within ASEAN’s information ecosystem, the hazard–exposure stream (AHA Centre Weekly Disaster Update and ADInet) is event-centric and geotemporal, describing flood intensity, extent, affected populations, and lifeline status; the ABVC dashboard is population- and administration-indexed, curating epidemic-prone syndromes; and wastewater/environmental surveillance contributes leading indicators of contamination and AMR pressure. Read as a single stack, these sources allow lead–lag analysis from inundation to environmental loading to clinical signal emergence, reconcile basin-scale impacts with district-level reporting, and surface risk concentration in low-lying informal settlements and agri-aquaculture corridors. Harmonizing on shared timestamps and spatial keys (administrative codes and river basins) preserves each platform’s native workflow while enabling One Health interpretation of flood-associated transmission dynamics across Member States. In the later sections of this review, we will explore these synergies with emerging techniques and integrative approaches.

## 2 From baseline to surge: microbial shifts in transmission

This review is guided by the DISEAASE framework, which Smith et al. (2022) addresses the compounded effect of disasters and epidemics, and how they increase human health risks. In SEA, flood-triggered outbreaks often involve multiple waterborne diseases shaped by overlapping geographical, socio-economic, pre-existing vulnerabilities and climatic drivers (Acosta-España et al., 2024). In operational terms, the DISEAASE framing maps cleanly onto One Health: disaster shocks perturb environmental reservoirs, alter animal–human contact, and intensify healthcare demand, so risk assessment must track all three domains together. Building on this framework, we examine flood-associated outbreaks in the region to draw lessons on disease transmission for future preparedness.

### 2.1 Pre-existing vulnerabilities increase risk of waterborne outbreaks

A combination of factors, such as climate change (heavy rainfall events, droughts), low elevation (coastal and river delta settlements), rapid urbanization (dense communities with inadequate drainage/sanitation systems), and socioeconomic impacts (poverty-stricken communities) are preexisting vulnerabilities that some AMS grapple with, increasing their susceptibility to floods and exposure to waterborne diseases and outbreaks (Fabrice et al., 2021). WHO data shows that unsafe water, sanitation, and hygiene (WASH) remain a major driver of global health burden, responsible for an estimated 1.4 million preventable deaths and 74 million disability-adjusted life years (DALYs). Although WHO reported global improvements in WASH and reduced vulnerability between 2015 and 2020, communities in LMICs, including those in SEA, remain highly vulnerable to climate change and limited access to safe drinking water, with persistent regional and intercountry disparities affecting local communities (Wen et al., 2025; Deshpande et al., 2020; Greenwood et al., 2024; World Health Organization, 2021). Many communities that live below the poverty line in SEA are experiencing regular floods (Rentschler et al., 2022), and are hit harder due to lack in WASH (World Health Organization, 2025b). Recent reviews show how these processes interact with environmental persistence, uncertain exposure pathways, and the challenges of managing risks across human, agricultural, and natural systems (Bolan et al., 2024; Crawford et al., 2022; Laino and Iglesias, 2025; Noyes et al., 2025; Uwishema et al., 2023; Zitoun et al., 2024).

### 2.2 Pathogen reservoirs in human settings

**Figure 1:**
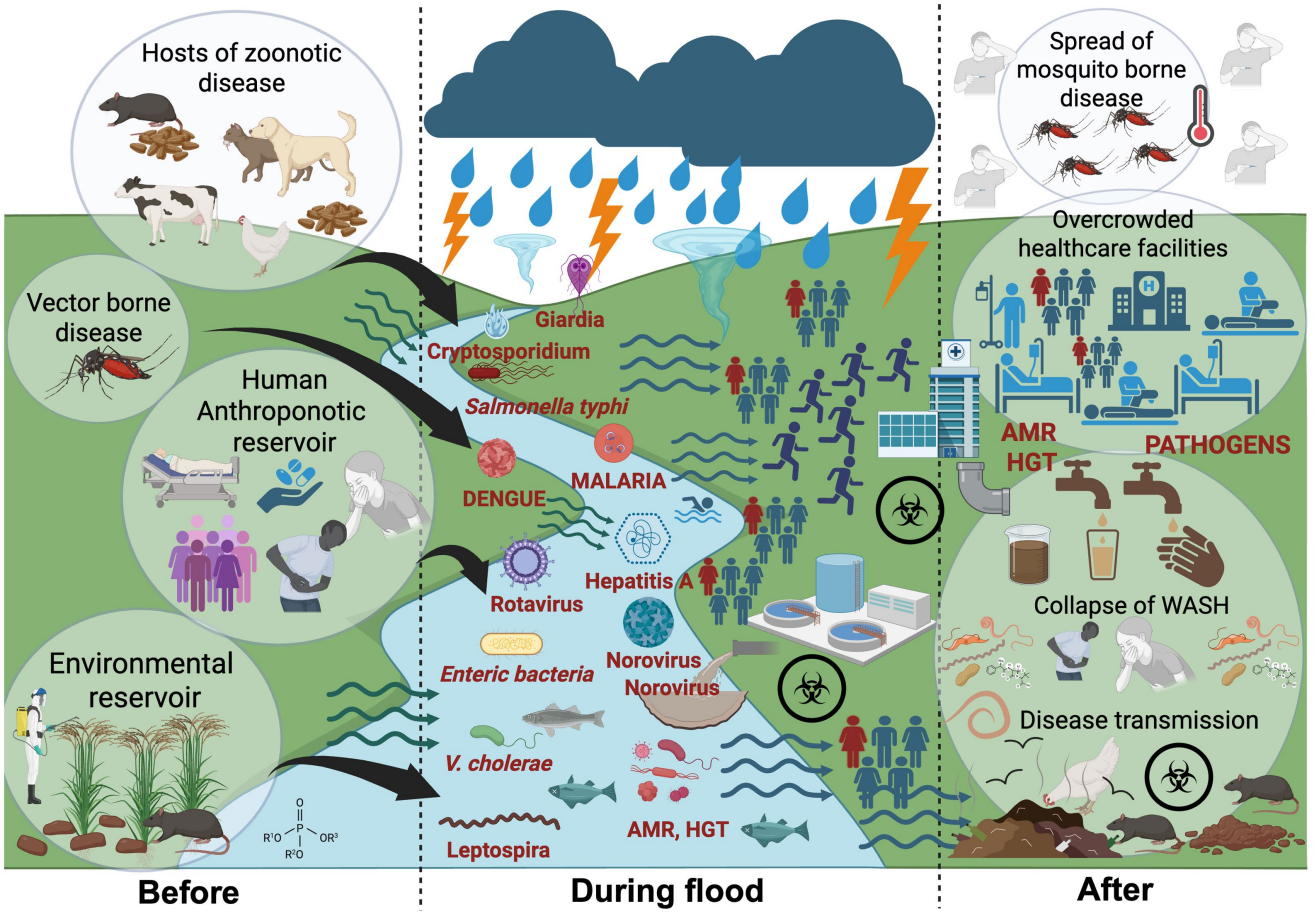
Transmission pathways of waterborne, vector-borne and zoonotic disease during major flooding events within a One Health context. Before floods: Zoonotic *Leptospira* spp. persists in environmental reservoirs such as waterlogged soils, paddy fields, and mud; maintained through zoonotic hosts including rodents, livestock, and stray animals. Other human-adapted pathogens (e.g., *Salmonella Typhi*, *Vibrio cholerae*, hepatitis A and parasite) circulate at low endemic levels in human populations, responsible for isolated or sporadic outbreaks. During floods: Storms, typhoons, and heavy rainfalls precipitate into major floods, dismantle ecological and sanitary barriers. Floodwaters mobilize pathogens through sewage overflows, fecal contamination, and runoff, while also driving zoonotic transmission by bringing animal pathogens into closer contact with humans. After floods: Stagnant water and blocked drainage sustain mosquito-borne diseases such as malaria and dengue. Failures in water, sanitation, and hygiene (WASH) systems fuel secondary outbreaks, while antimicrobial resistance (AMR) complicates treatment. Added pressures from physical injuries, overcrowding, and disrupted healthcare services further compound the public health burden. Created with BioRender.com.

Pathogen reservoirs in the environment and animal populations play a critical role in sustaining transmission between flood events (Figure 1). Among flood-related infectious agents, leptospirosis, caused by *Leptospira* spp., is the most commonly reported zoonotic disease worldwide. Leptospira comprises a diverse group of species and serovars, many of clinical relevance, and has been recently reviewed by Rajapakse et al. (2025) and Davignon et al. (2023). A comprehensive account of Leptospira serovars, case definitions, risk factors, epidemiological trends, and management strategies, specifically within the SEA context, has been recently reviewed by ASEAN Biodiaspora Virtual Centre (2024). More recent focused reviews of regional surveillance have also been published for individual AMS, including Malaysia (Ahmad Zamzuri et al., 2023; Azman et al., 2025; Philip and Ahmed, 2023; Lea et al., 2025), Thailand (Sawangpol et al., 2025) and Vietnam (Mai et al., 2022). Leptospira is endemic across SEA, with a wide range of animal hosts, causing isolated infections detected across all AMS between 2017 and 2023 (ASEAN Biodiaspora Virtual Centre, 2024). Rainfall frequency and intensity are commonly associated with heightened risk of leptospirosis. Infections often peak during periods of heavy rain and following flooding events (Chutinantakul et al., 2019; Matsushita et al., 2018; Phosri, 2022). Temporal data shows a rise of reported cases in Indonesia, Malaysia, Thailand, and Singapore over the past 3 years (2022–2025), while the Philippines saw a decline of cases after a 2018 peak, before rising again (ASEAN Biodiaspora Virtual Centre, 2024). Beyond public health, Leptospirosis also increases DALY and reduces global human productivity, resulting in significant global economic losses ($29.3 billion); with Indonesia, Thailand, and Malaysia reported as the three most affected AMS (Agampodi et al., 2023).

SEA with varied and diverse environmental niches such as rice paddies, wells, open water, and soils serve as environmental reservoirs for harboring dormant *Leptospira* that can reactivate under saturated wet conditions (Yanagihara et al., 2022). The pathogen has also been detected in contaminated surface waters, creating exposure risks through recreational and occupational activities (Narkkul et al., 2020; Narkkul et al., 2021). Populations living in rural areas and engaged in agriculture or farming practices are particularly vulnerable to infection (Sawangpol et al., 2025; Chadsuthi et al., 2021; Suttirat et al., 2025; Douchet et al., 2022), often occurring through direct contact with contaminated water or soil, when the skin is broken or abraded (Sakundarno et al., 2014). Within human settlements in urban and rural areas, zoonotic reservoirs play a major role in sustaining transmission, with high densities of commensal rodents consistently linked to infection risk (Sutiningsih et al., 2024; Widawati et al., 2023; Ribas et al., 2016; Villanueva et al., 2010; Ciano et al., 2021; Wuthiekanun et al., 2007; Krairojananan et al., 2025; Griffiths et al., 2022). Rodents are frequently found in environments such as wet markets and soils of paddy fields, where water samples from these areas test positive for Leptospira (Ling et al., 2025). In addition, exposure to stray animals (e.g., dogs, cats) (Narkkul et al., 2021; Chadsuthi et al., 2017; Ngasaman et al., 2020; Sprißler et al., 2019; Altheimer et al., 2020; Andityas et al., 2025) and livestock, (e.g., cattle, goat, sheep) (Sunaryo and Priyanto, 2022; Widiasih et al., 2021), carrying the pathogen, also represent a significant risk; as these animals can become hosts through infection after floods, or when exposed to contaminated water used for farming purposes (Lea et al., 2025; Widiasih et al., 2021; Rahman et al., 2023).

### 2.3 Anthroponotic reservoirs: source of sporadic outbreaks

Anthropogenic reservoirs of infection sustain a range of human-adapted pathogens that can fuel large diarrheal outbreaks following floods (Figure 1). Ingestion of fecally contaminated food or drinking water remains the primary route of transmission for a wide range of foodborne pathogens across AMS (Dewanti-Hariyadi, 2024). These pathogens including *Vibrio cholerae*, Enteric microorganisms, viral agents (e.g., norovirus, hepatitis A and E virus), and protozoan parasites (e.g., *Cryptosporidium*, *Giardia*) are often linked to sporadic outbreaks and isolated cases but can escalate into large-scale diarrheal events during floods (De Guzman et al., 2015; Lopez et al., 2015; Nga et al., 2018; Roberts et al., 2020; Alba et al., 2016; Muhammad et al., 2020; Swaddiwudhipong et al., 1995; Udompat et al., 2024). Like the other diarrheal pathogens mentioned earlier, typhoid caused by *Salmonella enterica* serovar Typhi also spreads through the fecal–oral route via contaminated water and food. Many of these pathogens including both *S. typhi* and toxigenic *V. cholerae*, linked to major outbreaks following a flooding event (Glass et al., 1984; Lopez et al., 2015; Simanjuntak et al., 2001; Roobthaisong et al., 2017; Karkey et al., 2016), can persist in aquatic environments and sediments during non-flood periods, especially when protected by organic matters (Liu et al., 2018; Tiwari et al., 2019; Eiler et al., 2007). The presence of these pathogens usually indicates the incidence of fecal contamination (Karkey et al., 2016; Oktaria et al., 2025). Viral agents also pose significant flood-related risks including waterborne viruses such as Hepatitis A and E, norovirus, and rotavirus, which are linked to outbreaks through contaminated food (Dewanti-Hariyadi, 2024), drinking and recreational waters (Mora et al., 2022). Wastewater, a major source of contamination during floods, can harbor a wide spectrum of clinically significant viruses such as enteroviruses, poliovirus, and SARS-CoV-2; many of which are now key targets of wastewater surveillance in several AMS (Pang et al., 2025). The presence of viruses such as SARS-CoV-2 in wastewater has been flagged as a potential public health risk during flooding events in urban settings (Han and He, 2021), although infectivity has not been conclusively demonstrated (Giacobbo et al., 2021). These findings underscore concerns of possible viral transmission through exposure to contaminated floodwaters.

Besides bacterial and viral etiologies, protozoan pathogens are an overlooked dimension to flood-associated disease risks. While some *Cryptosporidium* and *Giardia* species infect only humans, others are zoonotic and may be acquired through contact with animals, including pets (Ryan et al., 2021). In Asia and SEA, outbreaks caused by *Cryptosporidium* and *Giardia* are rare, accounting for less than 1% of all reported protozoan-related outbreaks (Bourli et al., 2023; Lim and Nissapatorn, 2017). Oocysts have been detected in surface waters, recreational and environmental sources, as well as in sewage (Lim and Nissapatorn, 2017; Masangkay et al., 2020) across several AMS, with low counts (0.06 ± 0.19 oocyst/L) also found in treated drinking water in the Philippines (Kumar et al., 2016). Practices such as open defecation, application of untreated human waste as fertilizer, and reliance on shallow wells (Nasim et al., 2022; Lam et al., 2015; Pham-Duc et al., 2013; Humphries et al., 1997) can keep pathogens in circulation. The global spread of the seventh cholera pandemic, traced by comparative genomic analysis to Sulawesi, Indonesia in 1961, illustrates how quickly such pathogens can proliferate once established (Luo et al., 2024). In effect, persistence of anthroponotic pathogens in both rural and urban settings can be sustained by human practices, with low-level transmission continuing until cross-contamination or flooding triggers large-scale outbreaks (Figure 1).

### 2.4 Changing climate and flood-induced shifts in transmission

**Figure 2:**
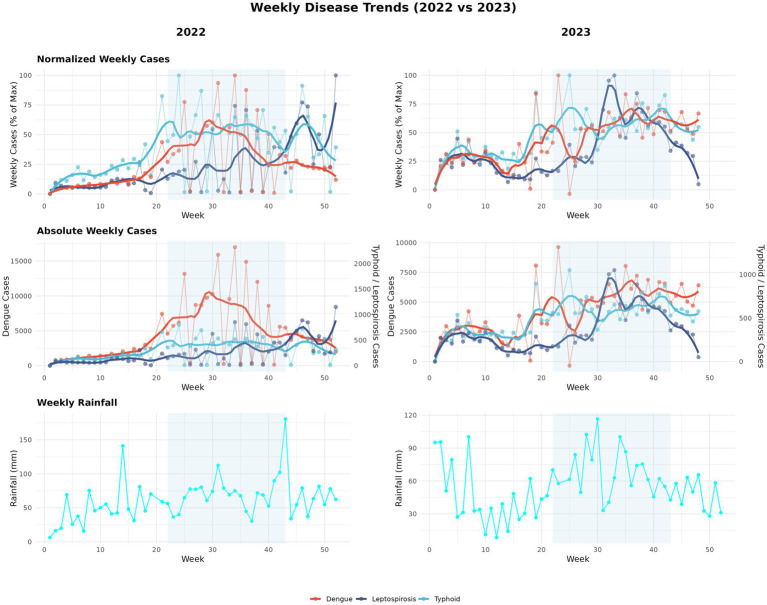
Seasonal dynamics of rainfall and disease cases in 2022–2023 (Philippines case study). Panels summarize a two-year case study for the Philippines. In 2023, rainfall was higher in January–February and peaked within the Southwest Monsoon window (June–October), whereas in 2022 the main rainfall maximum occurred later (≈epidemiological week 45). These timing differences correspond to earlier-season peaks in 2023 and greater persistence of weekly cases outside the monsoon window in 2022. Curves show weekly series with LOESS smoothing (span = 0.20) over raw values. Rainfall totals are weekly aggregates from daily CHIRPS and the Southwest Monsoon period (June–October) is shaded for reference. Epidemiological weeks and case counts are from national surveillance. Data from 2020 to 2021 were excluded due to COVID-19 lockdowns that affected routine reporting in the Philippines (Parreño, 2024). Rainfall: CHIRPS (Gorelick et al., 2017). Disease data: Department of Health, Weekly Disease Surveillance Reports (WDSR) (Department of Health- Philippines, 2025).

**Figure 3:**
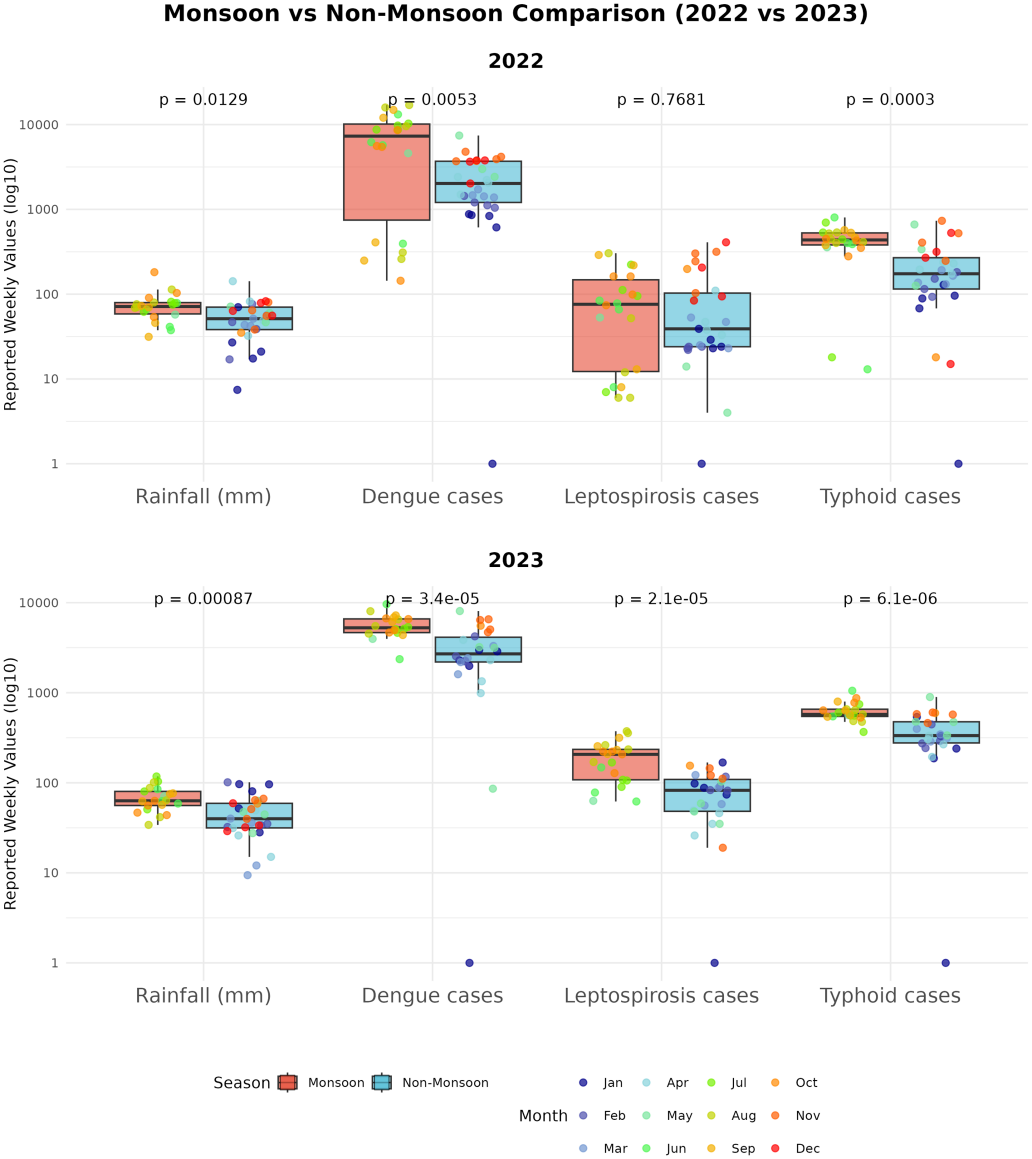
Monsoon vs non-monsoon comparisons (Philippines case study). Boxplots compare weekly rainfall and weekly new cases during monsoon (June–October) versus non-monsoon weeks for each disease and year; the y-axis is plotted on a log scale to place series of different magnitude on a comparable visual scale. Group differences were assessed with two-sided Mann–Whitney U tests (appropriate for skewed weekly counts); in our dataset, rainfall and all three diseases had significantly higher medians during monsoon weeks.

More than half (58%) of known human infectious diseases worsen with floods and other climate hazards, spreading through multiple transmission pathways that drive flood-related outbreaks (Mora et al., 2022). Most floods in SEA are driven by the East Asian Summer Monsoon (EASM), which brings peak rainfall during the boreal summer (May–September) (Chen et al., 2025; Loo et al., 2015). Seasonal monsoons are strongly associated with increased incidence of dengue, leptospirosis, and typhoid across several regions of SEA (Chadsuthi et al., 2021; Matsushita et al., 2018; Cunha et al., 2022; Benedum et al., 2018); for example, in Myanmar, peak rainfall has been linked to cholera outbreaks (Roobthaisong et al., 2017). Our Philippines case study (2022–2023) further illustrates this pattern (Figure 2). In 2023, rainfall was anomalously high during January–February and peaked within the core Southwest Monsoon (Weeks 22–43). This rainfall pattern coincided with elevated early-year dengue, leptospirosis, and typhoid case counts that declined soon after the monsoon subsided (Figure 3). In contrast, 2022 exhibited a late rainfall peak around epidemiological week 45, beyond the official monsoon window, and correspondingly sustained disease activity into the post-monsoon period. The persistence of cases beyond the monsoon season in 2022 is consistent with the delayed environmental effects of late-season rains (Chadsuthi et al., 2021; Matsushita et al., 2018; Cunha et al., 2022). To provide a balanced regional view, we tabulated flood-associated disease events across multiple AMS, including outbreaks following non-monsoon flood events, to illustrate the recurring post-flood transmission risk during and beyond the monsoon context (Table 1).

**Table 1:** Flood-related disease outbreaks in AMS between January 2024 to September 2025.

Date	Country/Province	Trigger	Disease/syndrome	Cases/Deaths	Corroboration	Source
Jan–Jun 2025	Thailand/North East Provinces	Monsoon, flood	Leptospirosis	1,623/20	Department of Disease Control	The nation (Public Health Ministry, n.d.)
Jun–Aug 2025	The Philippines	Monsoon, cyclone	Leptospirosis	4,859/unknown	Department of Health	Inquirer.net (DOH, n.d.)
Jan–Sep 2025	Indonesia/Riau Island, Aceh, Bangka Belitung	Sporadic heavy rains	Dengue	499/2 (Riau Islands) 108/unknown (Aceh) 200/unknown (Bangka Belitung)	Health authorities	The Jakarta Post (Asia News Network, n.d.)
Mar–May 2024	Indonesia/Central Java	Early-year floods	Leptospirosis	Over 200/unknown	Department of Health, Provincial	Antara (ANTARA News, n.d.), Jateng (PKS Jateng, n.d.)
Apr–May 2024	Indonesia/Pesisir Selantan, West Sumatra	Flash floods	Acute diarrhea	~200–300/5	Local Government	Antara (ANTARA News, n.d.), Pesisir Selantan (Kabupaten Pesisir Selatan, n.d.)
Nov 2024	Myanmar/ Yangon	Typhoon Yagi, WASH disruption	Acute watery diarrhea/cholera	205/unknown	WHO Regional Office for South-East Asia	WHO, 2024 (7th edition, 2024)
Dec 2024	Thailand/Tak Province	Rainy season floods (imported case)	Cholera	4/0	WHO Regional Office for South-East Asia	WHO, 2025 (Biweekly, n.d.)

With the impact of climate change, countries/regions including the Philippines, Singapore, Malaysia, Borneo, and Thailand are expected to see more intense rainfall, while Indonesia experiences fewer extreme events but longer stretches of consecutive wet days (Chen et al., 2025; Loo et al., 2015). Tropical cyclones are projected to persist longer and intensify, threatening key coastal cities such as Hai Phong, Bangkok, and Yangon, while extending damage farther inland (Garner et al., 2024). By the end of the century, SEA could see average regional warming of approximately 2 °C under moderate emissions (SSP245), rising to 3 °C or more under high emissions (SSP585), depending on the sub-region (Try and Qin, 2024). This mix of changing climates, complex surface and meteorological features means that monsoon-linked floods may become even more severe and unpredictable in the future.

Flooding from heavy rainfall, cyclones, or typhoons destroys natural and infrastructural barriers, facilitating contact between pathogens, vectors, and humans (Mora et al., 2022; Setiawati et al., 2022). Many pathogens persist at low endemic levels during non-flood periods and remain geographically contained until floods amplify transmission through dissemination into new regions previously unaffected (Ascione and Valdano, 2025; Bett et al., 2021). Floodwaters compromise sewage, and seed surface waters with enteric pathogens, mobilize pathogens from wildlife, livestock, human waste, and environmental reservoirs into shared aquatic systems (Mora et al., 2022; Chadsuthi et al., 2021; Rahman et al., 2023; ten Veldhuis et al., 2010; Thibeaux et al., 2024; Poulakida et al., 2024; Raya et al., 2024). Flood victims exposed to contaminated water are at high risk of infection via direct contact (e.g., skin abrasions) or through ingestion of water contaminated by sewage containing pathogenic organisms (Figure 1). Less commonly reported viruses further add to the burden, contributing through unknown pathways to post-flood illnesses such as respiratory diseases and skin infections (Mora et al., 2022). Compounded by inadequate WASH (Wen et al., 2025; World Health Organization, 2021) and intensified by climate change, heavy rainfall can devastate many areas across SEA; for example, recent seasonal rains in Myanmar in mid-2024 triggered yet another major cholera outbreak affecting 7,000 people and causing several deaths from contaminated water sources (World Health Organization, 2025a).

Beyond the immediate surge in waterborne infections, the timing and intensity of monsoon rainfall also govern the onset of vector-borne diseases. Standing water left behind after floods provides breeding grounds for mosquitoes, with dengue and malaria risks often peaking several weeks after the initial wave of diarrheal and leptospiral cases (Chadsuthi et al., 2021; Matsushita et al., 2018; Cunha et al., 2022; Benedum et al., 2018; Wang et al., 2023). This lag creates a critical window where health systems concentrate on waterborne outbreaks, while arboviral and parasitic threats accumulate more quietly in the background. Evidence from urban and national studies across the Philippines, Thailand, and Myanmar further shows that rainfall and flooding exert short-term, non-linear, lagged effects that elevate leptospirosis risk and amplify dengue transmission, underscoring how seasonal monsoons function as a recurrent driver of multi-disease dynamics in SEA (Chadsuthi et al., 2021; Matsushita et al., 2018; Cunha et al., 2022; Benedum et al., 2018). Frequent co-infections are reported in flood-affected regions including overlapping dengue-leptospirosis and melioidosis-leptospirosis cases (Lea et al., 2025; Asaduzzaman et al., 2024; Sachu et al., 2018). Animal reservoirs add another layer of complexity. In northern Vietnam, rodents were found to carry Rickettsia, Leptospira, and Bartonella at the same time (Anh et al., 2021), while rodents in Indonesian markets harbored both Leptospira and Orthohantavirus (Miura et al., 2025). Their role as reservoirs sustaining and spreading diseases across SEA has been comprehensively reviewed recently (Ganasen et al., 2025; Nguyen et al., 2025). Since most surveillance systems in ASEAN still monitor one disease at a time (Pang et al., 2025), such findings stress the risk of multi-pathogen spillover, which can delay recognition of overlapping outbreaks and lead to case misclassification.

## 3 Burden of floods on public healthcare

In the aftermath of a disaster, public health priorities in the emergency phase include ensuring access to food, shelter, health care, water supplies, sanitation facilities, control of communicable diseases, and public health surveillance (Wisner and Adams, 2002). There are three pathways by which floods can accelerate disease and death, (A) contamination of water supply which may lead to gastrointestinal illnesses and disease transmission, (B) stagnant breeding sites which harbor pests (e.g., rodents) and disease spreading vectors (e.g., mosquitos), (C) human displacement and poor access to sanitary conditions (Yang et al., 2024; Barbetta et al., 2022; Paterson et al., 2018; Suhr and Steinert, 2022; Lee et al., 2020). Compromised healthcare infrastructure associated with flooding events, and the sudden surge of patient care imposes a severe strain on healthcare facilities (Paterson et al., 2018). Risk of infection is a common cause of healthcare presentations after floods such as cutaneous and respiratory infections, and gastrointestinal, zoonosis and vector borne diseases as covered in the earlier sections of this review. A surge of noncommunicable diseases including chronic respiratory illnesses, cardiovascular disease, and diabetes emerge when disasters disrupt access to medical care and interrupt the supply of essential medication (McKinney et al., 2011; Ryan et al., 2015; Ryan et al., 2016). This is further compounded with acute health impacts such as orthopedic injuries, lacerations, hypothermia, electrocution, and burns that add to the public health burden during floods (Paterson et al., 2018). Drawing on data from over 300 million hospital admissions in 747 flood-prone communities, Yang et al. (2025) reported that floods were associated with elevated hospitalizations for infectious (RR = 1.26) and digestive diseases (RR = 1.30). These excess risks persisted for as long as 210 days after flooding, illustrating how the health impacts of floods extend well beyond the immediate crisis and reinforcing the need for sustained surveillance and preventive measures across SEA.

## 4 AMR as a disaster risk multiplier

AMR is a global public health priority, and it is projected that in 2050, 1.91 million annual deaths will be attributed to AMR worldwide (Naghavi et al., 2024). In the AMS region, sepsis accounted for ~4 million deaths in 2019, with 62% linked to bacterial infections. Of this, between 0.39 and 1.41 million deaths were attributed to AMR bacteria (Sihombing et al., 2023). LMICs are most susceptible to AMR spread due to misuse of antibiotics, availability of counterfeit drugs, fragmented health systems, inadequate/overcrowding of intensive care units (ICU) facilities, and inadequate WASH access (Rudd et al., 2018)particularly in times of crises. The two AMR indicators (*E. coli* resistant to third generation resistant cephalosporin (3GC), methicillin resistant *Staphylococcus aureus* (MRSA)) within the Sustainable Developmental Goals (SDG) monitoring framework showed slight overall improvement in bloodstream infections over 2017–2021; however countries such as Indonesia (70–75%), Nepal (65–77%) and Myanmar (77–84%) still face a fluctuating persistence of 3GC *E. coli* and other MDR pathogens (Sihombing et al., 2023).

Multiple intersections link climate change and extreme weather conditions to the emergence and spread of AMR, driven by the convergence of environmental contamination, intensive agriculture/farming practices, and rapid urbanization (Yasir Alhassan and Abdullahi Ahmad, 2025; Allel, 2021; Magnano San Lio et al., 2023; van Bavel et al., 2024). Within the One Health paradigm, these pressures emphasize the interconnectedness of human, animal, and environmental health, where AMR pathogens, and antibiotic resistance genes (ARGs) can circulate across ecosystem and amplify during climate related disruptions (Figure 1).

During and after disaster events (flooding, typhoons, earthquakes, tsunamis), displacement and overcrowding of hospitals, combined with the collapse of clean water and sanitation systems, and lack of medication, create optimal conditions for the silent spread of AMR and other communicable diseases (Noji, 2005). Damage to healthcare infrastructure often precipitates infection prevention and control (IPC) breakdowns, allowing resistant hospital-acquired infections to spread, while disrupted medical supply chains drive inappropriate or incomplete antibiotic use, intensifying selection pressure for AMR (Wisner and Adams, 2002; Skender and Zhang, 2024). Contamination of water systems from floodwaters carrying untreated sewage from damaged wastewater, hospital infrastructure, sewer overflows, agricultural runoff or industrial sites can introduce antibiotic resistant bacteria (ARB) and contaminants (e.g., disinfectants) that select for resistance (Furlan et al., 2024; Sethi et al., 2023). Aquatic environments that receive terrestrial effluents are AMR hotspots (ARB, ARGs) for horizontal gene transfer (HGT) between human and animal resistomes (Reverter et al., 2020). Another route of exposure for AMR transmission within a flood scenario is contamination of the food chain through aquaculture and livestock in flood-affected mariculture and agriculture areas that jeopardizes consumers of these food products (Furlan et al., 2024; Topp et al., 2018). Vietnam, Thailand, and Malaysia have the highest reports of AMR in aquaculture with *E. coli*, *Aeromonas*, *Vibrio* spp., being the most widely reported ARB, and tetracycline, beta-lactam, and sulpha the highest antibiotic classes detected in aquaculture across (Suyamud et al., 2024). Within SEA, few studies have documented pathogen-AMR disease (3GC resistance, MRSA, MDR) occurrences in human and environmental biomes following flooding events; however evidence remains scant, raising the need for strengthened surveillance (Table 2).

**Table 2:** Antimicrobial resistant bacteria detected after disaster events.

Disaster event	Samples	Country	Opportunistic pathogens	AMR	Reference
2004 Earthquake & tsunami	Multiple large-scale soft-tissue wound infections, respiratory infections of hospitalized patients	Across affected SEA countries	*Acinetobacter* spp., *E. coli*, *Staphylococcus aureus*, *Aeromonas hydrophilia*, *Pseudomonas* spp., *Candida albicans*	MDR *Acinetobacter* spp., ESBL *E. coli*, MRSA, MDR *A. hydrophilia*, MDR *Pseudomonas* spp., MDR *C. albicans*	Maegele et al. (2005)
2015 Flooding	Surface and groundwaters from Adyar river	Chennai, India	Coliforms, *Enterobacter aerogenes*, *Staphylococcus epidermis*, *Shigella flexneri*, *Streptococcus pyogenes*, *S. typhi, V. cholera*	Resistance to 3GC	Gowrisankar et al. (2017)
2022 Typhoon season	Mariculture tailwater treatment	Hainan, China	*Vibrio* spp., *Shewanella* spp., *Pseudomonas* spp., *Aeromonas* spp.	Beta-lactam resistant genes detected	Zhao et al. (2025)

Rising global temperature, erratic precipitation, and extreme weather events enhances microbial adaptation, bacterial survival, genetic mutations, and increases AMR transfer by HGT (Yasir Alhassan and Abdullahi Ahmad, 2025). *E. coli* and *Klebsiella pneumoniae* strains show a 4–5% increase in AMR with a 10 °C rise in temperature which is worrying in prolonged drought situations in SEA and reliance on unsafe water sources (MacFadden et al., 2018). Effective mitigation strategies including strengthening AMR surveillance and prediction models in the event of disasters would be beneficial to LMICs in SEA to curb the spread of AMR.

## 5 Surveillance of waste and surface water for early detection and monitoring

Floods often trigger outbreaks through sewage contamination. Wastewater surveillance targeting specific indicators offers an early-warning signal (Grassly et al., 2025; Hill et al., 2024). Within SEA, Singapore piloted the first wastewater surveillance to track SARS-CoV-2 during the COVID-19 pandemic (Ng et al., 2024; Martin et al., 2020; Wong et al., 2023); and antibiotic-resistant microorganisms and their removal from wastewater treatment plants (Ng et al., 2017; Ng et al., 2019; Li et al., 2022). Outside of SEA, the metagenomics of wastewater has revealed novel human-associated viruses (Bibby et al., 2019) and diverse parasites with potential clinical significance (Vatta and Cacciò, 2025). More recently, wastewater surveillance has garnered interest amongst ASM, with current efforts prioritizing five main targets: SARS-CoV-2, ARB, enteroviruses, influenza, and poliovirus (Pang et al., 2025). WHO’s Wastewater and Environment Surveillance (WES) framework (World Health Organization, 2024), still in a pilot phase, has already issued guidelines for cholera, with other targets; influenza, monkeypox, polio, and COVID-19; while *S. typhi*, *Leptospira*, and other waterborne viruses are pending finalized guidance. Lessons from early adopters like Singapore could accelerate the integration of WES to include flood-associated pathogens in AMS in the future.

For surveillance of surface waters and environments, studies outside of AMS have demonstrated the use of eDNA metabarcoding to track pathogenic *Leptospira* in aquatic ecosystems, correlating their presence with rainfall trends and vertebrate markers (Sato et al., 2019; Sato et al., 2025; Sato et al., 2024; Gamage et al., 2020; Sato et al., 2022; Sato et al., 2025). On the clinical side, research has demonstrated the value of using metagenomics for earlier leptospirosis diagnosis in patients (Jiang et al., 2022; Li et al., 2024; Han et al., 2024; Ji et al., 2023). When integrated with epidemiological and One Health data streams, omics can significantly strengthen global health surveillance (Hill et al., 2024; Aßmann et al., 2025; Koutsoumanis et al., 2019). Shotgun metagenomics of gut microbiomes combined with machine learning (ML) has identified microbial and functional biomarkers predicting *V. cholerae* susceptibility and disease severity (Levade et al., 2020). More recently, Zhuang et al. (2025) applied ML to metagenomic sequencing of multiple sewage sources, enabling detection of emerging SARS-CoV-2 variants days to weeks ahead of clinical reporting and the identification of novel mutation signatures. Together, these advances demonstrate the value of combining ML and omics approaches for early disease signal detection in wastewater and highlight their potential to expand beyond SARS-CoV-2 toward a broader panel of waterborne and vector-borne pathogens relevant to flood-prone regions.

## 6 Conclusion

Climate change is driving more frequent and intense rainfall across SEA, and more severe floods are expected in the coming decades. This review has examined pathogen persistence, transmission, and the public health impacts of floods. These risks interact with other climate-related factors. Typhoons, rising temperatures, algal blooms, and changes in freshwater lakes all influence access to clean water. Higher temperatures also affect the survival and growth of waterborne pathogens, many of which are not directly linked to human sources. Floods amplify the spread of pathogens and accelerate their distribution. They also increase the circulation of ARBs, placing heavy pressure on already burdened local hospitals. The WHO WASH program has achieved progress over the last decade. Yet outbreaks such as cholera in Myanmar and persistent challenges in mainland SEA, Indonesia and the Philippines show that these improvements remain fragile under climate stress. Flood-associated health risks require a One Health approach that links human, animal, and environmental health. Advances in metagenomics, omics technologies, wastewater surveillance, and machine learning can support predictive systems to anticipate outbreaks. Alignment and application of these tools will strengthen surveillance and coordinated regional action. ASEAN, through its disaster response and health security platforms, can play a pivotal role in connecting member states, sharing knowledge, and building capacity. Delivering this vision requires formalizing One Health incident playbooks with shared indicators, joint action thresholds, and co-funded monitoring across health, agriculture, and environment so that floods trigger coordinated rather than siloed responses. Taken together, the AHA Centre Weekly Disaster Update, ADInet, ABVC, and wastewater/environmental surveillance constitute an ASEAN-specific One Health information stack in which hazard, health, and environmental signals are co-registered in time and space for flood-associated outbreak analysis. Working in unison will help SEA build climate-resilient health security and strengthen preparedness for future flood-related disease threats.
